# Antimicrobial Peptides in Preventive Medicine: Current Perspectives on Coating Strategies

**DOI:** 10.1021/acsinfecdis.5c01050

**Published:** 2026-02-24

**Authors:** Milan Wouters, Laurence Van Moll, Emine Derin, Sara Van Looy, Linda De Vooght, Peter Delputte, Paul Cos

**Affiliations:** † Laboratory of Microbiology, Parasitology and Hygiene (LMPH), Faculty of Pharmaceutical, Biomedical and Veterinary Sciences, 26660 University of Antwerp, Wilrijk, Antwerp 2000, Belgium; ‡ Department of Basic and Applied Science, University of Basilicata, Potenza 85100, Italy

**Keywords:** device-associated infections, antimicrobial peptides, antibacterial coatings, preventive medicine

## Abstract

The alarming rise of antimicrobial resistance and the declining efficacy of conventional antibiotics emphasize the need for preventive strategies. Within the setting of device-associated infections, antimicrobial peptides (AMPs) have been extensively studied as antimicrobial candidates, owing to their broad-spectrum activity and structural versatility, enabling integration into functional surface coatings. This review provides a comprehensive overview of AMP-based prophylactic approaches, with a particular focus on coatings for medical devices prone to biofilm formation, such as endotracheal tubes, catheters and implants. While surface immobilization of peptides can be accomplished through comparatively straightforward methodologies, the field has progressed toward sophisticated matrix-based systems that enhance stability, biocompatibility and controlled functionality. Yet, despite extensive in vitro and small-scale in vivo studies, clinical translation remains very limited and constrained by several hurdles including regulatory ambiguity and production costs. Overall, this work aims to provide an up-to-date overview of the AMP-based technologies in infection prevention research.

## Graphical abstract

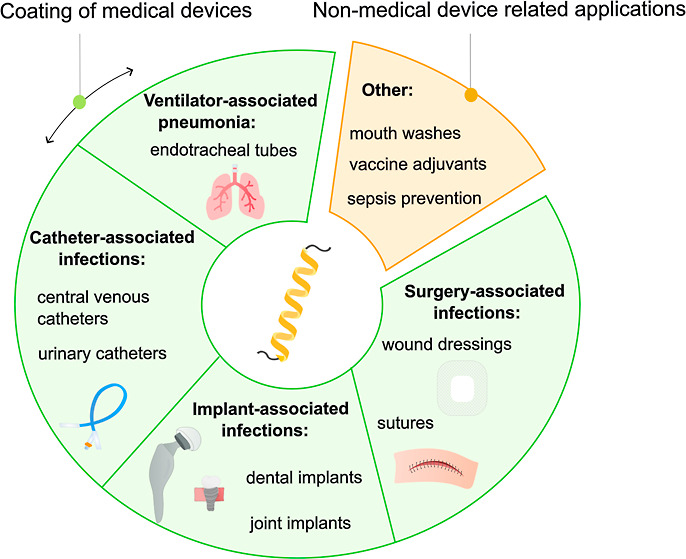

## 1 Introduction

With the rise of antimicrobial resistance (AMR), many drugs routinely used in clinical practice are failing to treat critical infectious diseases.^1^ Tackling this AMR emergency requires a multifaceted approach, including the discovery and development of new antimicrobial agents that are active against drug-resistant pathogens.^2^ Antimicrobial peptides (AMPs), a diverse family of small, evolutionary conserved peptides, have attracted growing attention as alternatives to the conventional antibiotics on the market due to their pleiotropic activities, including direct microbial killing, immunomodulation and role in tissue repair.^3^ AMPs are found all throughout the kingdoms of life, including animals, plants and lower life forms such as prokaryotes.^4^ As effector molecules of the innate immune system, AMPs are also referred to as “host defense peptides”.^5^ With their cationic and amphipathic character, AMPs usually exert their antimicrobial activity by interacting with the negatively charged microbial membranes, followed by the disruption of the membrane bilayer integrity, which leads to a potent and fast microbicidal effect.^5^ Due to this fast mechanism of action, AMPs exhibit a stronger maximum killing effect than conventional antibiotics, displaying a shorter window in which resistant mutants can emerge.^6^ Moreover, AMPs can simultaneously act on multiple microbial targets, decreasing the chance of pathogens developing full resistance against the peptides.^7^ Overall, their potent and broad antimicrobial spectrum and lower propensity for resistance development have made AMPs promising candidates for therapeutic development.

Despite the widespread interest in AMPs, their development as new antibiotics has been hindered by several challenges. Their poor druggability, limited in vivo stability and bioavailability, and weak correlation between in vitro efficacy and in vivo performance have significantly complicated their clinical translation.^7^ In addition, several AMPs have been withdrawn from clinical trials due to unexpected toxic side effects such as renal failure.^8,9^ Consequently, development of AMPs has mostly focused on applications with localized delivery, as challenges related to oral bioavailability, stability and systemic toxicity can be largely circumvented.^7^ Next to the local use of AMPs in the treatment of infectious diseases (e.g., wound infections), the use of AMPs in preventive medicine holds significant promise. Prophylactic measures are essential in lowering the health and economic burden of infectious diseases, as well as in decreasing the emergence and spread of AMR. Research and development of AMP-based infection prevention strategies have primarily focused on peptide-based coatings for medical devices to reduce the risk of device-associated infections (DAI). DAIs are a leading cause of hospital-acquired infections (HAI), contributing to prolonged hospital stays, increased healthcare costs and elevated morbidity and mortality rates.^10,11^ AMP coatings could positively impact the healthcare workspace, limiting infections such as ventilator-associated pneumoniae (VAP), catheter-associated urinary tract infections (CAUTI), catheter-related bloodstream infections (CRBSI), implant-associated infections (IAI) and surgery-site infections (SSI), all depicted in Figure 1. Beyond hospital and medical device applications, research on prophylactic AMPs use is much more limited, focusing primarily on dental care, particularly the development of mouthwashes to prevent oral infections and dental caries. In addition, the immunomodulating properties of AMPs are of interest in infection prevention. Exploratory research on AMPs in sepsis prevention investigates their endotoxin-binding properties, and their immunomodulatory activity is of interest in vaccine development as well. Overall, these emerging applications highlight the potential of AMPs beyond conventional antimicrobial therapies, paving the way for innovative strategies in infection prevention.

**Figure 1:**
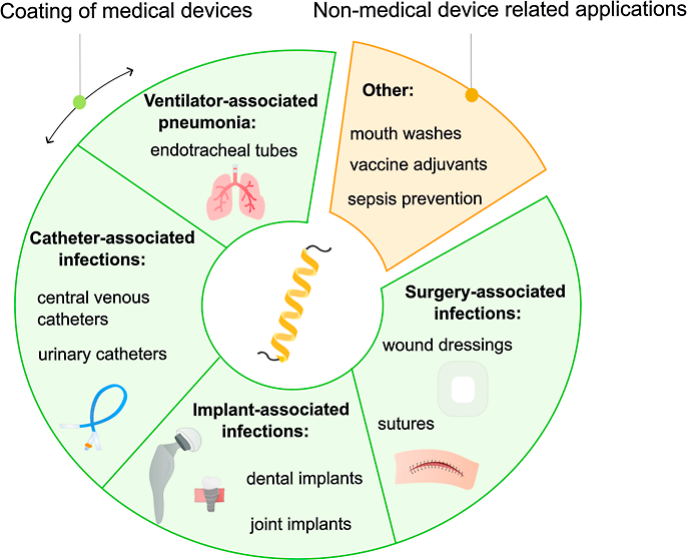
Graphical overview of potential applications of antimicrobial peptides in preventive medicine. Currently, the largest research field is the development of antimicrobial peptide-based coatings for medical devices, including endotracheal tubes, urinary and venous catheters, implants, wound dressings and sutures. Outside of coatings, antimicrobial peptides are researched as adjuvants in mouth washes or vaccines.

## 2 Scope

This narrative review explores the various strategies by which AMPs can be deployed prophylactically to prevent microbial infections, particularly focusing on AMP coatings in the frame of DAIs. Emphasis was placed on naturally occurring gene-encoded AMPs, their semisynthetic truncated or chemically modified derivatives and fully synthetic AMP-like peptides. Therefore, commercially available lipopeptides and glycopeptides (e.g., vancomycin, colistin, daptomycin) were excluded, as these agents are typically reserved as last line therapeutics. Furthermore, this review exclusively addresses AMP strategies with a primary role in infection prevention, excluding applications with other goals such as wound healing.

A literature search, based on the PRISMA guidelines, of the PubMed database was conducted using pathology-specific search strings, as detailed in Supporting Information Section S1. Two reviewers independently screened titles, abstracts and full-text articles retrieved from PubMed according to the following eligibility criteria: (i) prophylactic use of AMPs (excluding therapeutic applications), (ii) inclusion of all AMPs except commercially available lipopeptides and glycopeptides, and (iii) incorporation of the AMP into a coating on a medical device. For every selected study, five key characteristics were extracted, organized and summarized in DAI-specific tables below: publication year and author, type of DAI, peptide specifications, coating strategy and substrate type. This review adopts a narrative approach supported by a PubMed literature search, with the objective of critically synthesizing current knowledge in the field and identifying unmet needs and opportunities for future research. All records were managed in Mendeley Reference Manager (London, United Kingdom).

## 3 Peptide Coating Strategies for Antibacterial Surface Design

When exploring AMPs for prophylactic applications, antimicrobial coatings represent the most investigated strategy to prevent the onset and/or progression of bacterial infections. These coatings can be categorized into antifouling coatings and active antibacterial coatings.^12^ Antifouling strategies typically do not involve the incorporation of membranolytic AMPs and are primarily designed to prevent microbial adhesion to abiotic surfaces.^13^ Although various strategies have been developed, steric hindrance combined with surface hydration is the most explored mechanism.^14,15^ While early efforts mainly focused on the incorporation of synthetic polymers, with polyethylene glycol (PEG) being the gold standard, recent research on this topic is dominated by zwitterion-containing polymers on bioinspired materials.^14,16^

Besides antifouling surfaces, active antibacterial coatings incorporating AMPs exist. As proposed by Drexelius et al., active antimicrobial coatings can be categorized into three distinct subtypes based on their mode of peptide incorporation, as shown in Figure 2.^17^ Primary coatings involve the direct application of AMPs onto the surface of the medical device, either through immersion in AMP solution or via covalent attachment using specific binding sequences within the peptide.^17^ Secondary coatings introduce an intermediate linking layer, such as polydopamine (PDA) or silanization, that facilitates adhesion of AMPs to the substrate.^17^ Lastly, tertiary coatings embed AMPs within degradable or porous matrices that permit controlled and sustained release over time, thereby extending the duration of antimicrobial activity at the site of application.^17^

**Figure 2:**
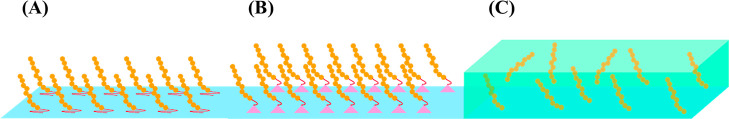
Different coating subtypes with (A) primary coating (B) secondary coating containing an intermediate linking layer (pink triangles) and (C) tertiary coating of the peptide loaded into a matrix. Figure based upon Drexelius et al.^17^

Both the primary and secondary approaches involve peptide immobilization onto the surface, enabling them to eliminate or inhibit bacteria upon direct contact. Such surfaces are commonly referred to as contact-killing surfaces, as they exert their antimicrobial effect upon interaction with bacterial cells in proximity.^18^ For these contact-killing coatings, AMPs can be immobilized using physical methods, such as the layer-by-layer (LbL) assembly technique which is based on electrostatic interactions between cations and anions. Nevertheless, chemical immobilization via covalent linkage is preferred due to its superior stability and long-term efficacy.^19^ AMPs have been functionalized onto various substrates such as plastics, metals and titanium; however, several challenges remain.^19,20^ Peptide distance from the surface, orientation and mobility after tethering all critically affect peptide activity.^18,19^ It is well established that the effective antibacterial concentration of AMPs increases when they are tethered to a surface, likely due to reduced mobility and limited access to bacterial membranes.^19,20^ Furthermore, Lou et al. demonstrated that peptides immobilized via their C-terminus exhibited enhanced interactions with bacterial membranes. This was attributed to the positively charged N-terminus remaining exposed and readily available to engage with the negatively charged bacterial membrane.^21^

In addition to contact-active surfaces, AMPs can also be incorporated into controlled-release coatings, where AMPs are embedded within a matrix that enables sustained diffusion over time.^17^ The majority of extended-release coatings are based on polymeric hydrogel-based systems, which provide a cross-linked hydrophilic network that can encapsulate and gradually release its active component over time.^22–24^ A wide range of polymers has been investigated for AMP delivery, with chitosan and gelatin representing leading candidates among natural polymers, while poly­(vinyl alcohol) (PVA) and poly­(lactic-*co*-glycolic acid) (PLGA) are the most commonly used synthetic polymers.^22,25^ By incorporating AMPs into a polymeric network, covalent attachment to the material surface becomes unnecessary and as a result, the peptide backbone can remain unmodified and the use of linkers is not required.^17^ Hydrogel networks are typically characterized by their biocompatibility, adjustable mechanical properties and nontoxic degradation products.^25,26^ Although each polymer possesses inherent limitations, these drawbacks can be addressed by selecting the polymer most suited to the intended application. Synthetic polymers are often preferred over their natural counterparts due to their high degree of tunability, enabling precise control over properties such as degradation rate, mechanical strength and release kinetics. This tunability offers possibilities to alter and control the rate of drug release. For example, increasing coating thickness prolongs AMP release and adjusting pore size can facilitate the incorporation of less water-soluble AMPs.^22,27^ These principles have driven the development of stimuli-responsive hydrogels, which can reversibly alter properties as wettability, charge or morphology in response to external stimuli.^12^ Relevant stimuli include physical cues (e.g., temperature, light, etc.), biological triggers (e.g., cells or enzymes) or chemical alterations in the microenvironment (e.g., pH, ionic strength, etc.).^12,28^ In the context of anti-infectious applications, pH-responsiveness and enzyme-triggered responsiveness are among the most explored mechanisms.^22,29–31^ The former being attributable to the acidic microenvironment generated at the site of infection and the latter referring to bacterial secondary metabolites.^31,32^

Next to hydrogel networks, other strategies like polymer brushes or titanium nanorods exist, but are explored to a lesser extent.^17^ These strategies respectively encompass the linking of an AMP to polymer brushes tethered to the device’s surface or the storage and subsequent release of AMPs from pockets made via an electrochemical reaction at a titanium surface.^17^

### 3.1 Ventilator-Associated Pneumonia

Ventilator-associated pneumonia (VAP) is a nosocomial infection characterized by inflammation of the lung parenchyma that arises in patients undergoing mechanical ventilation.^33,34^ It is among the most critical DAIs, affecting up to 20–37% of critically ill patients in intensive care units (ICU) with mortality rates varying between 24 and 76%, depending on underlying risk factors.^10,33^ VAP is primarily caused by either streptococci, in the case of early onset VAP, or multidrug resistant (MDR) Gram-negative bacteria such as *Pseudomonas aeruginosa* and *Klebsiella pneumoniae* in the case of late-onset VAP.^33,35–37^ Less frequently, late-onset VAP may be attributed to Gram-positive pathogens such as *Staphylococcus aureus*, or in rare cases, to fungal organisms.^35,36^

Endotracheal tubes (ETTs) used in the clinic are commonly made from polyvinyl chloride (PVC) and are mostly uncoated. Available commercial coatings are nonpeptidic and offer only moderate benefits. Silver-coated ETTs reduce and delay VAP but do not improve ICU stay, ventilation duration, or mortality.^38^ Additionally, the Bactiguard ETT, based on a silver–palladium–gold alloy, is still under development and not Food and Drug Administration (FDA)-approved.^39,40^

To date, limited research has been published on the use of peptide-coated ETTs for the prophylaxis of VAP. The performed PubMed search only registered one hit according to the previous mentioned eligibility parameters, namely the work of Zhu et al., who incorporated FK13-a1, a truncated fragment of a human cathelicidin, into a hydrogel on the surface together with the drug Meropenem.^41^ This coating prevented bacterial colonization on the tube surface in a rat VAP model, with the combination coating showing stronger antibacterial activity than either single-agent coating. In addition, its pH-responsiveness enabled controlled release under more acidic pH, potentially reducing side effects in healthy tissue. No additional literature on the use of true gene-encoded AMPs for VAP prevention was identified; however, Wouters et al. developed a zwitterionic hydrogel coating containing the lipopeptide polymyxin B on PVC ETTs.^42^ The peptide had an extended release for 42 days and inhibited biofilm formation on the tube’s surface. Other studies have investigated ceragenins for VAP prevention. Ceragenins are cationic steroid antimicrobials with a mechanism of action that mimics that of endogenous AMPs.^43,44^ Their rapid bactericidal activity, coupled with a synthetic origin and a sterol-based backbone that confers resistance to proteolytic degradation, makes them attractive candidates for incorporation into antimicrobial coatings.^43^ Lastly, Aronson et al. developed a coating for ETTs incorporating the antimicrobial peptide lasioglossin-III. Although the coating provides sustained release and effectively reduces airway infections along the ETT, it was specifically designed to prevent subglottic stenosis and was not intended to address VAP.^45^

### 3.2 Urinary-Tract Infections

Catheter-associated urinary tract infections (CAUTIs) occur when an indwelling urinary catheter introduces bacteria into the urinary tract and gives rise to a bladder infection maximum 48 h after catheterization. Outside of the ICU, CAUTIs represent the most common HAI and the most frequent cause of secondary bloodstream infections.^46^ Clinically, CAUTIs can present with symptoms such as fever, suprapubic or flank pain, hematuria and changes in urine appearance or odor. If left untreated, CAUTIs can lead to complications such as pyelonephritis and bacteremia. The infections usually arise from the patient’s own rectal flora, including uropathogenic *Escherichia coli*, *K. pneumoniae*, *Proteus mirabilis*, *P. aeruginosa*, or *Enterococcus species*, but other causative pathogens including *S. aureus* and *Candida* species are encountered as well.^46,47^ A wide variety of urinary catheters are available on the market, with polymer substrates consisting of either silicone, latex, rubber, polyurethane or PVC and offered in both uncoated or coated forms (e.g., hydrogel or Teflon-coated).^47,48^ As antibacterial options, only noble metal-coated urinary catheters are available on the market.^48,49^ They may reduce bacteriuria during short-term use in adults; however, the impact on symptomatic CAUTI remains unclear due to low-quality evidence.^49^

As of 2014, peptide-based urinary coatings have been progressively explored in scientific studies (Table 1). Recent research demonstrates a strong focus on antibacterial coatings with a Gram-negative spectrum targeting primarily *E. coli* and *P. aeruginosa*, or broad spectrum with additional antistaphylococcal activity. In a study by Raman et al., a strictly antifungal coating using AMPs was developed for urinary catheters.^50^ Although yeasts like *Candida albicans* are frequently isolated from the urine of patients presenting with CAUTI (up to 15% of infections), the clinical significance of candiduria in catheterized patients and its causative relation to CAUTI remain controversial, thereby justifying the focus on antibacterial coatings.^46,47^ Despite the AMP’s activity being localized to the catheter surface, functional performance depends on stability within the bladder environment. Coating stability is therefore usually tested in artificial urine media. Urine constitutes a unique physiological matrix, with AMP coatings needing to withstand varying pH’s (4.5–8), high ionic strength, various metabolites (e.g., urea) and proteases.^51^ In addition to linear peptides, other strategies to obtain physiologically stable AMPs can be considered. White et al., for example, developed the AMP CD4-PP, a cyclic KR12 (LL-37 derivative) dimer with increased stability in urine and improved activity against uropathogens, which prevented *E. coli* from adherence to urinary catheters.^52^

**Table 1:** Overview of AMP-Based Coating Strategies for Urinary Catheters^a^

coating type	AMP	AMP sequence	material	ref
Primary: anionic surfactant facilitated coating	SAAP159	LKRLYKRVFRLLKRY YRQLRRPVR	silicone	^54^
	RRIKA	WLRRIKAWLRRIKA	silicone	^54^
Primary: peptide immersion	Rilk1-Cat	RLKWVRIWRR	silicone	^53^
Secondary: thiol-maleimide coupling of peptide to PEG-AGE polymer brushes	CysLasio-III	VNWKKILGKIIKVVK	silicone	^60^
Secondary: AGE polymer brushes	RK1	RWKRWWRRKK	silicone	^62^
	RK2	RKKRWWRRKK	silicone	^62^
Secondary: polydopamine	CWR11	CWFWKWWRRRRR	silicone	^57^
Secondary: thiol-maleimide coupling of peptide to polymer brushes	E6	RRWRIVVIRVRR	PU	^58^
Secondary: polydopamine–polymer composite coating	E6	RRWRIVVIRVRR	PU	^59^
Secondary: polydopamine–polymer composite coating	TET20-LC	KRWRIRVRVIRK-bA-bA-C	PU	^59^
Secondary: polydopamine	MP196 derivative	RWRWRW	PU	^55,56^
Secondary: silanization	PEP-C, C-PEP	RLLLRLLRRLLRLLLR	silicone	^61^
Tertiary: polyelectrolyte multilayer film	β-peptide	[(ACHC-β^3^hVal-β^3^hLys)]_3_	PE	^50^
Tertiary: unspecified polymeric matrix	CP11-6A	KKLIKKILKIL	silicone	^63^
Tertiary: PCL and POPC polymeric matrix	HHC36	KRWWKWWRR	silicone	^65^
Tertiary: PEG–PCL copolymer matrix	HHC36	KRWWKWWRR	silicone	^64^
Tertiary: PVP polymeric matrix	CD4-PP	KRIVQRIKDFLRKRIVQRIKDFLR (two KR-12 peptides cyclized)	polyolefin-based elastomer pre-coated with PVP	^52^

a Abbreviations: AGE, allyl glycidyl ether; PCL, poly-ε-caprolactone; PE, polyethylene; POPC, 1-palmitoyl-2-oleoyl-*sn*-glycero-3-phosphocholine; PU, polyurethane; PVP, polyvinylpyrrolidone.

Given the diversity of materials used in commercially available urinary catheters, strategies for formulating peptide-based coatings are equally varied. Most research so far has focused on silicone and polyurethane catheters, as these materials are the most widely used in clinical practice. Primary coatings, either using immersion techniques or surfactant-driven coatings, have been explored by Giaquinto et al. and Wang et al.^53,54^ As PDA can adhere effectively to virtually any biochemical, it is a commonly used linker to bind bioactive molecules such as AMPs in secondary CAUTI coatings.^55–57^ PDA, however, is also prone to nonspecific interactions with human proteins, leading to decreased coating efficacy over time. To address this limitation, Yu et al. designed a substrate-independent dual coating by coassembling PDA with high molecular weight hydrophilic polymers.^58,59^ Next to PDA-based coatings, other secondary strategies explored for CAUTI prevention include coupling AMPs to catheter surfaces via silanization or covalently binding the peptides to brush polymers.^58–62^ As tertiary coating strategies, various polymeric matrix coatings have been investigated, including a PEG–PCL (poly-ε-caprolactone) copolymer that enable controlled release.^50,63,64^

### 3.3 Catheter-Related Bloodstream Infections

Catheter-related bloodstream infection (CRBSIs) can occur when pathogens contaminate central venous catheters (CVCs) extra- or intraluminally. Although CRBSIs have the lowest prevalence among HAIs, reported at 0.4% according to the European Centre for Disease Prevention and Control (ECDC), the associated morbidity and mortality remain clinically significan.^11^ Notably, CRBSIs have a relatively high incidence outside of the ICU, in contrast to most other HAIs. In 2022, over 50% of reported CRBSI cases occurred in general inpatient wards, mostly due to patients requiring prolonged venous access outside the ICU, such as individuals receiving hemodialysis or cancer treatment.^66^ The microbiology of CRBSI is highly variable across regions, time periods and patient populations. Historically, skin-associated Gram-positive pathogens such as *S. aureus* predominated CRBSIs.^67^ More recent surveillance, however, shows a shift toward Gram-negative bacteria and *Candida* species, with increasing multidrug resistance. Studies report fungal CRBSI in up to 24% of cases and MDR pathogens in nearly half, particularly among Gram-negative infections and long-term catheter use.^68–70^ In the clinic, CVCs can be impregnated with conventional antibiotics and antiseptics.^71,72^ The most widely used coatings include impregnation with minocycline/rifampicin, chlorhexidine/silver sulfadiazine and rifampicin/miconazole.^71,73^ Although these coatings have been associated with reduced CRBSI rates, this has not yet translated into a significant reduction in mortality.^71^ CVCs coated with agents such as 5-fluorouracil, gendine, levofloxacin, *N*-acetylcysteine and teicoplanin are also under investigation; however, their clinical use remains limited due to lower efficacy compared with established impregnation strategies.^73,74^ Additionally, noble metal-based coatings have also been explored and are clinically available for CVCs.^75^

Although peptide-coated CVCs were introduced over two decades ago, their development has seen limited progress in recent years. Bower et al. were the first to incorporate the AMP nisin onto PVC tubing using a primary immersion approach. To date, only a limited number of additional studies have reported on the incorporation of AMPs onto CVCs to prevent CRBSIs (Table 2).^76^ Recent approaches show increasing innovation in peptide immobilization strategies. Notable examples include the secondary strategy using PEG linkers to ensure spatial conformation stability, as reported by Berglin et al., and the tertiary, hierarchical multilayer systems that combine antifouling and bactericidal functionalities, as described by Zhang et al.^77,78^

**Table 2:** Overview of AMP-Based Coating Strategies for Central Venous Catheters^a^

coating type	AMP	AMP sequence	material	ref
Primary: peptide immersion	Nisin	I{DHB}AI{DHA}LA{ABA}PGAK{ABA}GALMGANMK{ABA}A{ABAKAHASIHV{DHA}L	PVC	^76^
Secondary: plasma immersion ion implantation	Melimine	TLISWIKNKRKQRPRVSRRRRR	PVC	^79^
	Mel4	KNKRKRRRRRRGGRRRR	PVC	^79^
Secondary: silane assisted SI-ATPR coupling of peptide to multi-carboxylic polymer brushes	HHC36	KRWWKWWRR77	PU	^77^
Secondary: polydopamine	mPEP, cPEP	/	silicone	^80^
Secondary: various click chemistry mechanisms	AMC-25-04	/	PU	^78^
Tertiary: polyelectrolyte multilayer film	β-peptide	[(ACHC-β^3^hVal-β^3^hLys)]_3_	PE	^50^

a Abbreviations: PE, polyethylene; PVC, polyvinyl chloride; PU, polyurethane; SI-ATPR, Surface-initiated atom transfer radical polymerization.

Lastly, CVCs can also be treated with catheter lock solutions (CLS) as a strategy to prevent catheter-related infections. These solutions are instilled into the catheter lumen and left in place for a defined dwell time between uses. Traditionally, CLS contained heparin to reduce the risk of thrombosis. However, more recent approaches have incorporated antimicrobial agents, most notably antibiotics such as aminoglycosides, as adjuncts to inhibit bacterial colonization and biofilm formation within the catheter.^81,82^ Although not surface-bound, CLS containing AMPs have been explored as an alternative strategy to prevent infection. Cirioni et al. were among the first to investigate AMP-based CLS, evaluating the efficacy of the cathelicidin BMAP-28.^83^ Subsequently, Zapotoczna et al. examined the use of the synthetic AMP Bac8c for *S. aureus* biofilm inhibition.^84^ Despite these promising findings, AMP-based CLS remain in the early stages of development and even well-established CLS formulations containing clinically validated antibiotics are currently reserved as adjunct therapies for specific high-risk patient populations rather than for routine use.

### 3.4 Implant-Associated Infections

Implant-associated infections (IAIs) are infections attributable to medical devices implanted in the body, such as cardiac valves, stents and dental or orthopedic implants.^85,86^ The use of biomedical devices in the clinic is rising and IAIs currently represent over one-quarter of all HAIs in the US.^87^ In the context of prophylaxis against DAIs, IAIs represent the most critical target for prevention. Unlike temporary catheterization tubing which can be removed in the event of infection, implanted medical devices typically cannot be easily explanted. Consequently, effective strategies to prevent IAIs are of paramount importance. Causative bacteria are predominantly opportunistic pathogens derived from the patient’s skin microbiota. Gram-positive staphylococci such as *S. aureus* and *S. epidermidis* are the most common pathogens, though other organisms such as *P. aeruginosa* and *Candida* or *Cryptococcus* species also contribute to IAIs.^86^ Microbial biofilm formation on the device leads to local inflammation and infection of the surrounding tissues or organ, and in severe cases, can result in systemic dissemination via the bloodstream. IAIs can occur days postimplantation, or, in some cases, months postoperatively due to the formation of an abscess or sinus.^88^ Ultimately, IAIs can lead to surgery failure and the need to remove the implanted device.^85^ Commercially available silver- or antibiotic-coated orthopedic implants exist; however, their clinical adoption in Europe remains limited due to reimbursement challenges and concerns regarding cytotoxicity and immunogenicity.^89^

To date, AMP coatings have been predominantly investigated in the context of orthopedic and dental implants (Table 3), which are associated with a general infection risk of 5 and 14% respectively.^90^ Although associated with the highest incidence of bloodstream infection after implantation, cardiac implants (e.g., valves or electronic devices) have not yet been the focus in AMP research.^91^ Coating of implant surfaces poses a considerable technical challenge, as strong antifouling surfaces can slow the healing process by preventing cells and tissue from adhering. AMPs exert not only microbial killing but also promote tissue regeneration such as osteogenesis, osseointegration and angiogenesis. Accordingly, peptide-based coatings are often designed to serve this dual function. To enhance cellular adhesion, AMPs for implant coatings can be engineered to include the RGD (arginine–glycine–aspartic acid) motif, a well-known cell-binding sequence that interacts with integrin receptors on host cells.^92–94^

**Table 3:** Overview of AMP-Based Coating Strategies for Implants^a^

coating type	AMP	AMP sequence	material	ref
Dental implants				
Secondary: silanization	GLK13	GKIIKLKASLKLL	titanium	^101^
Secondary: silanization	unnamed	KKKGGGGRGDS	zirocinia/zirconia-Titanium	^92^
Secondary: silanization	Pac-525	Ac-KWRRWVRWI	titanium	^127^
Secondary: polydopamine	d-amino K122-4		titanium alloy nanotubes	^102^
Secondary: silanization and thiol-maleimide coupling	Cys-GLK13	CGKIIKLKASLKLL	titanium	^106^
Tertiary: polyelectrolyte multilayer film	Tet231-collagen, renamed AMPcol	KRWWKWWRRC	titanium	^110^
Tertiary: composite and graphene oxide matrix	Nal-P-113	Ac-AKR-Nal-Nal-GYKRKF-Nal	titanium	^128^
Tertiary: polyelectrolyte multilayer film	unnamed	lauryl-VVAGK-Am	stainless steel/glass	^111^
Tertiary: polydopamine + titanium oxide nanotubes	LL-37	LLGDFFRKSKEKIGK EFKRIVQRIKDFLRNL VPRTES	titanium	^117^
Tertiary: PCL polymeric matrix	caerin 1.9 (F3)	GLFGVLGSIAKHVLPHVVPVIAEKL	titanium	^129^
Orthopedic implants				
Primary: peptide immersion	Tet231	KRWWKWWRRC	titanium	^96^
Primary: metal-binding sequence	unnamed	LKLLKKLLKLLKKL	titanium	^97^,^98^

Primary: peptide immersion	Mel4	KNKRKRRRRRRGGRRRR	titanium	^95^
Secondary: covalent binding via thiol groups	unnamed RGD containing peptides	/	collagen-coated titanium	^93^
Secondary: polydopamine	cecropin B	KWKVFKKIEKMGRNIRNGIVKAGPAIAVLGEAKAL	titanium	^130^
Secondary: polydopamine	KR12	KRIVQRIKDFLR	PEEK	^100^
Secondary: polydopamine	unnamed	Phe_10_-*stat*-Lys_12_	titanium	^109^
Secondary: EDC coupling of peptide to graphene oxide layer on sulfonated PEEK	Nisin	FY(Dha)LGK4NLDCVKLGNTCPIPGF(Dha)VFKVNNKFVAK	SPEEK	^105^
Secondary: polydopamine	Dhvar5	LLLFLLKKRKKRKY	titanium	^103^
	MSI78	GIGKFLKKAKKFGKAFVKILKK		
Secondary: silanization	KR12	KRIVQRIKDFLR	titanium	^131^
Tertiary: collagen and methoxysilane matrix	human β-defensin-2	GIGDPVTCLKSGAICHPVFCPRRYKQIGTCGLPGTKCCKKP	titanium	^132^
Tertiary: calcium phosphate matrix	HC36	KRWWKWWRR	titanium coated with calcium phosphate	^115,116^
Tertiary: calcium phosphate matrix	Tet231	KRWWKWWRRC	titanium coated with calcium phosphate	^116^
Tertiary: hydroxy apatite matrix	PSI 10	RRWPWWPWRR	magnesium alloy coated with hydroxyapatite	^133^
Tertiary: gelatin hydrogel	HC36	KRWWKWWRR	titanium alloy	^134^
Tertiary: bone cement matrix	HAL-1	GMWSKILGHLIR	poly(methyl methacrylate)	^114^
	HAL-2	GKWMSLLKHILK		
Tertiary: bone cement matrix	H27/H27D	GKWMKLLKKILK	poly(methyl methacrylate)	^112^
	H29/H29D	GKWVKLLKKILK		
Tertiary: immersion in PEG slurry	unnamed AMP from snails	/	titanium alloys, titanium-stainless steel and polyethylene	^135^
Tertiary: chitosan nanogel	Dhvar5	LLLFLLKKRKKRKY	/	^136^
Tertiary: silk fibroin-based composite film incorporating lysozyme and ZIF-8 nanoparticles	Pt5-1c	SAMLLTALIIGLTALTHLLATLAHHSATL	titanium	^137^
General implants				
Primary: spray coating	unnamed	he(4F)-Phe(4F)-Arg-Gly-Asp	PDMS	^94^
Primary: peptide immersion	GLK13	GKIIKLKASLKLL	titanium	^138^
Primary: peptide immersion + surface etching	SHAP1	APKAMKLLKKL LKLQKKGI	titanium, PCL	^139^
	P5	YIRKIRRFFKKLK KILKK		
Secondary: covalent attachment via cysteine	hlF1-11	GRRRRSVQWCA	chitosan thin films	^107^
Secondary: EDC coupling of peptide substrate	GLK13	KIIKLKASLKLL	PEEK	^104^
Secondary: polydopamine	Polylysine	lysine polymer	titanium	^140^
Tertiary: titaniumoxide nanotubes	GLK13	GKIIKLKASLKLL	titanium	^118^
Tertiary: calciumphosphate matrix	AMP	KRWWKWWRR	titanium coated with calcium-phosphate	^113^
	cHABP1-AMP	CMLPHHGAC-GGG-KRWWKWWRR		
Contact lenses				
Primary: immersion of lenses in peptide solution	melimine	TLISWIKNKRKQRPRVSRRRRRRGGRRRR	hydrogel	^141^
Secondary: EDC coupling	melimine	TLISWIKNKRKQRPRVSRRRRRRGGRRRR	hydrogel	^120,124,141^
			silicone	^121,124^
Secondary: EDC coupling	Mel4	KNKRKRRRRRRGGRRRR	silicone	^123^
			hydrogel	^119,142^
			hyaluronic acid-laden hydrogel	^122^
Secondary: EDC coupling or alkyn-azido covalent coupling	IG-25	IGKEFKRIVQRIKDFLRNLVPRTES	fluorosilicon	^126^
Secondary: polydopamine coating	B4010	(RGRKVVRR)4	hydrogel	^143^
Secondary: EDC coupling or via oxazoline plasm or plasma ion immersion	TM5, TM18	Ntridec-NLys-Nspe–Nspe-NLys, Ndec-(NLys-Nspe-Nspe)2-NLys)	hydrogel	^125^

a Abbreviations: EDC, 1-ethyl-3-(3-dimethylaminopropyl)­carbodiimide; PEEK, polyetheretherketone; PCL, poly-ε-caprolactone; SPEEK, sulfonated polyetheretherketone.

Research on AMP coatings for IAIs has explored a variety of functionalization strategies, primarily applied to titanium-based implants. Primary immersion coatings have been explored by Zhang et al. and Zhao et al.^95,96^ To enhance the peptide binding affinity and orientation, some researchers have incorporated solid-binding sequences (e.g., titanium-binding domains) into the peptide backbone.^97,98^ These engineered motifs facilitate efficient self-assembly of the AMP to the metal oxides without the need for additional linking layers and long, complex coating chemistry.^99^ However, these solid-binding peptides can be prone to in vivo instability due to competition from proteins in biological fluids and proteolytic degradation.^99^ Designing new peptides or fusion-peptides with solid-binding domains also poses a significant technical challenge compared with the use of unmodified AMPs.

Similar to CAUTI and CRSBI, secondary coatings are the most popular in the development of antimicrobial implant devices, with the functional intermediary scaffolds often being PDA or silanes.^92,100–103^ While the PDA technique has been applied to both polymer- and metal-based implants, silane-based methods remain largely confined to titanium surfaces. This is likely because polymers lack reactive hydroxyl groups, requiring surface activation prior to silanization, increasing process complexity.^17^ Secondary strategies exploiting functional groups of AMPs to establish coatings include EDC-mediated coupling, which have been investigated by Kumar et al. and Hu et al. for polyetheretherketone (PEEK) implants.^104,105^ Hu et al. achieved direct peptide immobilization on unmodified PEEK via oxime ligation, exploiting its native ketone groups without requiring surface activation.^104^ In contrast, Kumar et al. used EDC chemistry to conjugate nisin to sulfonated PEEK.^105^ Additionally, AMPs functional groups can be modified to induce a substrate-peptide binding.^93,106,107^ For example, in Schliephake et al., binding to collagen-coated titanium was induced via thiol anchor groups.^93^ Similarly, Costa et al. describe the immobilization of hLF1-11 to collagen thin films via the terminal cysteine residue.^107^ The addition of linkers in the AMP sequence can improve peptide mobility and increase the accessibility of the antimicrobial domain. In Maddikeri et al. and Costa et al., PEG linkers were used in the coating strategy, while Liu et al. explored a DOPA linker fused to an antimicrobial polypeptide.^107–109^ Tertiary coating strategies for IAIs explore the integration of AMPs into advanced delivery systems, such as TiO_2_ nanotubes, LbL matrices, hydrogel systems and bone cement.^110,111^ Bone cement, used to anchor orthopedic implants to the bone, can be composed of either polymers (poly­(methyl methacrylate)) or calcium phosphate-containing compounds. Both types of bone cement have been investigated as carriers for AMPs, either through covalent binding or loading the peptide in the matrix.^112–116^ Similarly, TiO_2_ nanotubes promote osteoblast adhesion and stem cell differentiation by mimicking the nanostructure of hydroxyapatite and have been explored as carriers for the AMPs as well by Li et al. and Zhang et al.^117,118^

Lastly, AMP coated contact lenses have also been explored by several research teams. Although not traditional implants, contact lenses are often worn for extended periods and in case of weekly or monthly contact lenses, repeated use and exposure to human skin increase the risk of microbial keratitis. Coating strategies have mostly focused on covalently binding the AMP to the contact lens surface via EDC coupling. Mel4 or melimine coated hydrogel lenses have been studied extensively, up to in vivo infection studies and human clinical trials.^106,119–124^ Other research has investigated fluorosilicone lenses with the LL37 derivative IG-25 and hydrogel lenses modified with TM5 and T18 peptides.^125,126^

### 3.5 Surgical Site Infections

Surgical site infections (SSIs) represent the most common postoperative complications and are typically classified into three categories: superficial incisional, deep incisional and organ/space infections.^144^ According to the latest report from the European Centre for Disease Prevention and Control, skin infections account for a substantial 23.9% of all HAIs, ranking third in prevalence.^10^Within this category, soft tissue infections constitute nearly 70%, with implanted medical devices posing a particularly high risk due to the extended postoperative window of up to one year, during which infection of surrounding tissues may still occur.^10^ These concerning figures are further supported by the substantial economic burden of SSIs, which can exceed $90,000 per case when associated with prosthetic joint implants.^145^

SSIs are predominantly caused by endogenous flora, with *S. aureus* being the principal organism.^146,147^ Notably, methicillin-resistant *S. aureus* (MRSA) accounts for over 10% of *S. aureus*-related SSIs, with prevalence reaching as high as 30% in the context of orthopedic surgeries.^146–148^ Although Gram-negative bacteria are generally less prevalent in SSIs, *E. coli* remains the leading cause of infections following colorectal or abdominal surgery.^146,147^ Despite not having the highest prevalence or morbidity, antimicrobial coatings for wound dressings and sutures are commercially abundant. Unlike many other medical devices, these products are consumables requiring frequent replacement, which drives market volume and profitability.^149^ Additionally, most coated wound dressings and sutures fall under medical device classes I or II, allowing for less demanding and less costly regulatory pathways as they are primarily positioned for prevention rather than treatment.^150^ Compounds such as metal nanoparticles, iodine, biguanides, chitosan or even peptides are regularly incorporated and used in clinical practice.^149^ Peptides loaded in wound dressings; however, are limited to established lipo- or glycopeptides such as daptomycin, gramicidin or vancomycin.^149,151^ The range of antimicrobial-coated sutures is limited; however, triclosan-coated versions are WHO-endorsed with grade B evidence for reducing SSIs.^152,153^

Within the domain of AMP coatings for wound dressings and sutures, a variety of coating strategies has been researched (Table 4). Only a few primary coatings have been studied, including HNP-1 dip-coated sutures and drop-casted wound dressings using melittin-derived peptide 1.^154,155^ The secondary strategies for SSIs are again highly diverse and include, for example, polylysine coated sutures using maleimide–thiol click chemistry and PEG nanofibers, and AMP spin-coating on benzophenone functionalized polyurethane wound dressings.^156,157^

**Table 4:** Overview of AMP-Based Coating Strategies for Sutures and Wound Dressings^a^

coating type	AMP	AMP sequence	material	ref
Sutures				
Primary: dip coating	hyperbranched polylysine	/	PGA	^166^
Primary: dip coating	HNP-1	ACYCRIPACIAGERRYGTCIYQGRLWAFCC	silk fibroin	^154^
Secondary: cold atmospheric plasma conjugation	unnamed peptide	KRFRIRVRV	PGCL	^167^
	unnamed peptide	RWRWRWRW	PGCL	^167^
Secondary: maleimide–thiol click chemistry to PEG nanofibers	K18 (poly lysine)	/	silk fibroin, PGA and PGLA	^157^
Wound dressings				
Primary: drop-casting on MECs	melittin-derived peptide 1 (MDP1)	GIGAVLKVLTTGLPALIKRKRQQ	/	^155^
Secondary: covalent immobilization using “Sulfo-SAND”	lysozyme, lysostaphin, HBD-3, LL-37	/	polypropylene	^168^
Secondary: spin-coating on a benzophenone-functionalized substrate	synthetic mimics of antimicrobial peptides (SMAMPs)	/	PU	^156^
Secondary: chitosan film with a SM(PEG)8 spacer arm	MSI-78 (4-20)	KFLKKAKKFGKAFVKIL	/	^169^
Secondary: mesoporous silica with a BODIPY linker	C14R	CSSGSLWRLIRRFLRR	/	^160^
Secondary: immersion of plasma-treated electrospun PCL nanofibers	Nisin	/	/	^170^
Tertiary: polyelectrolyte multilayer	ponericin G1	GWKDWAKKAGGWLKKKGPGMAKAALKAAMQ	silicone	^171^
Tertiary: Ffmoc-protected d-phenylalanine hydrogels	C14R	CSSGSLWRLIRRFLRR	/	^161^
Tertiary: hyaluronic acid-based hydrogel	Polyarginine	/	/	^159^
Tertiary: recombinant silk with antimicrobial motifs as engineered nanofibrous mats or microporous scaffolds	Magainin I	GIGKFLHSAGKFGKAFVGEMKS	silk fibroin	^172^
	Lactoferricin	FKCRRWQWRMKKLGAPSITCVRRAF	silk fibroin	^172^
Tertiary: polyelectrolyte multilayer + Polydopamine	KR-12	KRIVQRIKDFLR	eggshell membrane nanofibres	^158^
Tertiary: hyaluronic acid macroporous hydrogel	DP7 (in silico origin)	VQWRIRVAVIRK	/	^173^

a Abbreviations: MECs, methacrylated chitosan; PEG, polyethylene glycol; PGA, polyglycolic acid; PGCL, poly­(lactic-*co*-glycolic) acid; PU, polyurethane.

Unlike applications targeting CRBSI, IAI, or CAUTI, tertiary strategies predominate in wound dressings. This preference likely stems from the intrinsic material properties of wound dressings, typically porous and (semi)­hydrophilic substrates such as cotton or polyurethane, which offer enhanced substrate compatibility. For instance, Liu et al. engineered a LbL assembly incorporating KR-12 on a hydrophilic eggshell membrane, while Gribova et al. embedded a polyarginine peptide within a hydrogel matrix on a cellulose mesh.^158–162^

Nonetheless, Table 4 provides only a limited selection of AMPs focusing on SSIs prevention, since the research in the field predominantly prioritizes the treatment of established infections, with secondary emphasis on promoting wound healing and angiogenesis following the onset of SSIs. When antimicrobial compounds are incorporated into these strategies, the focus often lies on the use of broad-spectrum, high-potency agents, such as vancomycin or colistin, primarily aimed at therapeutic intervention rather than prophylaxis.^163^ Although many peptides possess dual functionality, their primary application often centers on wound healing, with infection prevention considered a secondary benefit. While having multiple immunomodulatory functions, peptides such as human cathelicidins or β-defensins can act as chemoattractant to guide immune cells to the wound site or can suppress neutrophil apoptosis.^164,165^ The incorporation of these peptides contributes not only to combating existing infections, but also to accelerating the healing of acute or chronic wounds.

### 3.6 Current Landscape of AMPs and Coating Types

Antimicrobial coatings utilize a wide variety of peptide candidates, though distinct design trends can be identified. Synthetic AMPs are particularly favored, either created de novo through rational engineering or inspired on natural scaffolds with targeted modifications that enhance their antimicrobial activity or coating compatibility. Computational tools such as machine learning are being used increasingly to accelerate the discovery and optimization of AMPs. For example, HHC36, a 9 amino acid (AA) synthetic AMP generated with the aid of neural network-based design, has been used in the development of coatings for IAI, CAUTI and CRBSI.^64,65,77,116,134^ Beyond computational design, peptide length represents another critical parameter that is frequently optimized in synthetic AMPs. While most natural AMPs are classified as long (>30 AA), synthetic designs often favor intermediate (13–30 AA) or short length (7–12 AA) sequences (Figure 3). Ultrashort AMPs (<7 AA) are cost-effective and easily modifiable antimicrobial motifs that combine synthetic accessibility with potential for high stability; however, they are less commonly used compared to short or intermediate peptides, possibly because their minimal length can compromise antimicrobial efficacy and strain selectivity.^50,55,56,78,93,94,111,125^

**Figure 3:**
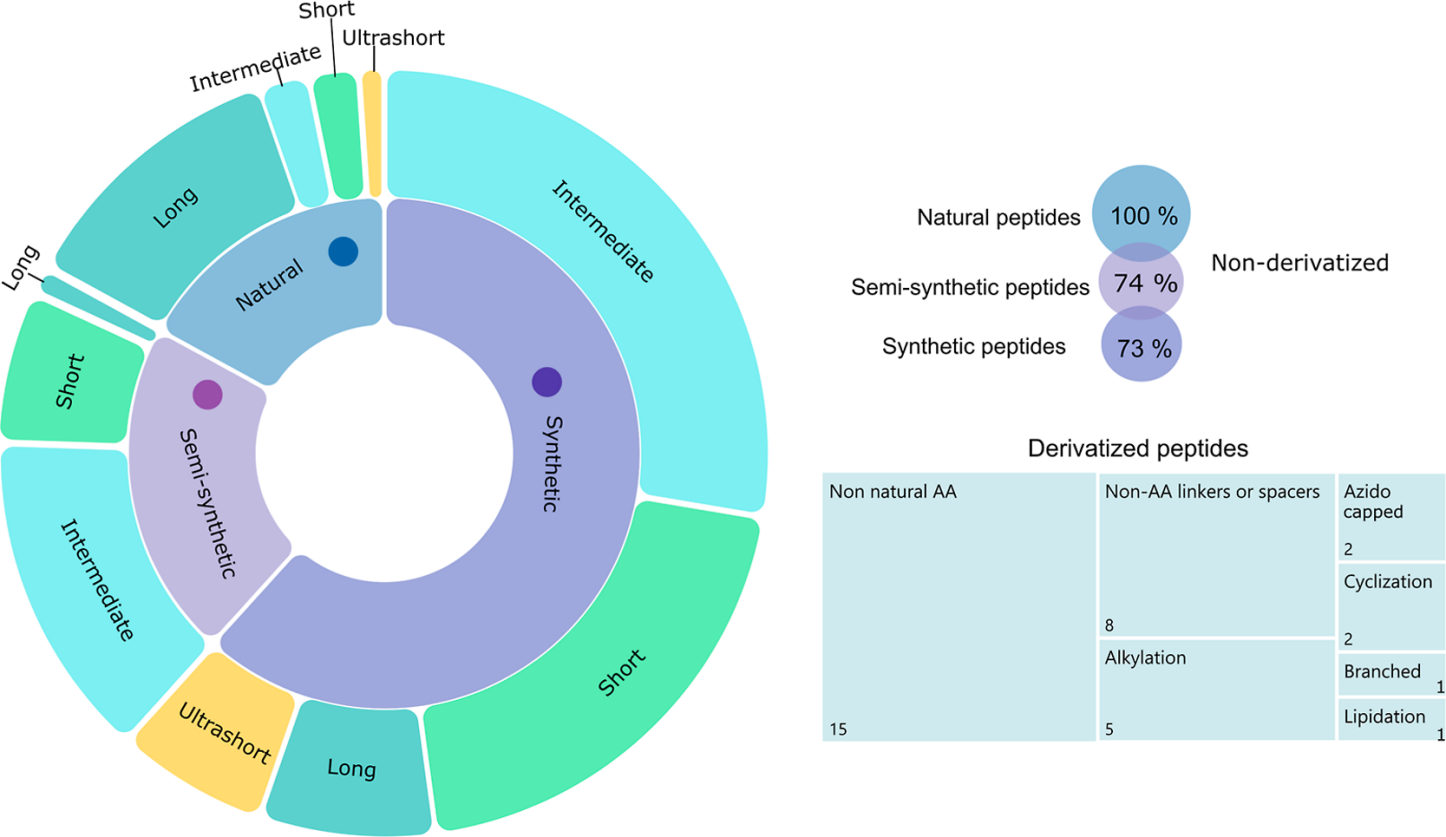
Overview of the characteristics of antimicrobial peptides investigated for medical device coating. Left: Most peptides are synthetic, either entirely novel or loosely inspired by natural templates. While interest in ultrashort peptides (<7 AA) is increasing, the majority fall within short (7–12 AA) or intermediate (13–30 AA) length ranges. Right: Most synthetic and semisynthetic peptides are nonderivatized; among derivatized variants, the inclusion of non-natural AA and chemical linkers or spacers is most common.

Widely studied natural AMPs and templates for (semi)-synthetic design include cathelicidins, notably the human cathelicidin LL-37, human defensins such as β-defensin-2 and β-defensin-3, salivary proteins including histatins and salivary secretory proteins, as well as tsehe bee venom peptide melittin. In semisynthetic AMP development, shortening or truncation of natural sequences is a common strategy to generate shorter analogs that are easier to synthesize, more stable and frequently retain equal or enhanced antimicrobial activity with reduced off-target effects. For example, KR12 represents the minimal active fragment of LL-37, while IG-25 is derived from LL-37 via truncation.^100,126,131,158^ Simple AA substitutions or extensions are frequently employed to optimize activity or adapt peptides for integration into coatings. A typical modification is the introduction of terminal cysteine residues, which facilitate covalent attachment to coating surfaces through thiol-based chemistry.^58–61,106,110,115,136^ Peptide derivatization represents another optimization strategy, involving chemical modifications beyond AA residue substitution, such as cyclization, alkylation, or terminal capping (Figure 3). Among the different derivatization strategies, the most common are the incorporation of non-natural AA (e.g., D-AA or α- or β-substituted AA), which improve proteolytic stability and allow fine-tuning of charge or hydrophobicity and the use of chemical linkers or spacers (e.g., PEG), which facilitate peptide presentation and flexibility on surfaces.^50,58,78,80,93,94,98,107–109,112,126,131,139,156,169^ Other modifications include alkylation, to enhance membrane interactions, as well as cyclization or azido capping, which can be used to stabilize the AMP structure or enable covalent coupling respectively.^50,52,78,80,125,126^

### 3.7 Translational Progression of AMP-Based Coatings

The body of research on AMP-based antimicrobial coatings has expanded substantially, complicating the identification of approaches that are both innovative and translationally relevant. Figure 4 contextualizes the literature discussed in this narrative review by mapping translational maturity across clinical pathologies, specifying the developmental stage and the coating type used. Despite the predominance of in vitro studies, several groups have progressed preventive AMP-based coating strategies to small or large in vivo investigations. Animal models are associated with well-recognized limitations, including difficulties in achieving infection chronicity, the use of clinically unrealistic inoculum sizes and challenges in establishing mature biofilms.^174–176^ Nevertheless, these animal models provide whole-body complexity and multifactorial biological responses that can never be obtained in simplified in vitro systems.^174,175^ This complex system remains to date a crucial step in bridging the gap between early in vitro data and clinical studies of new antimicrobial medical devices.

**Figure 4:**
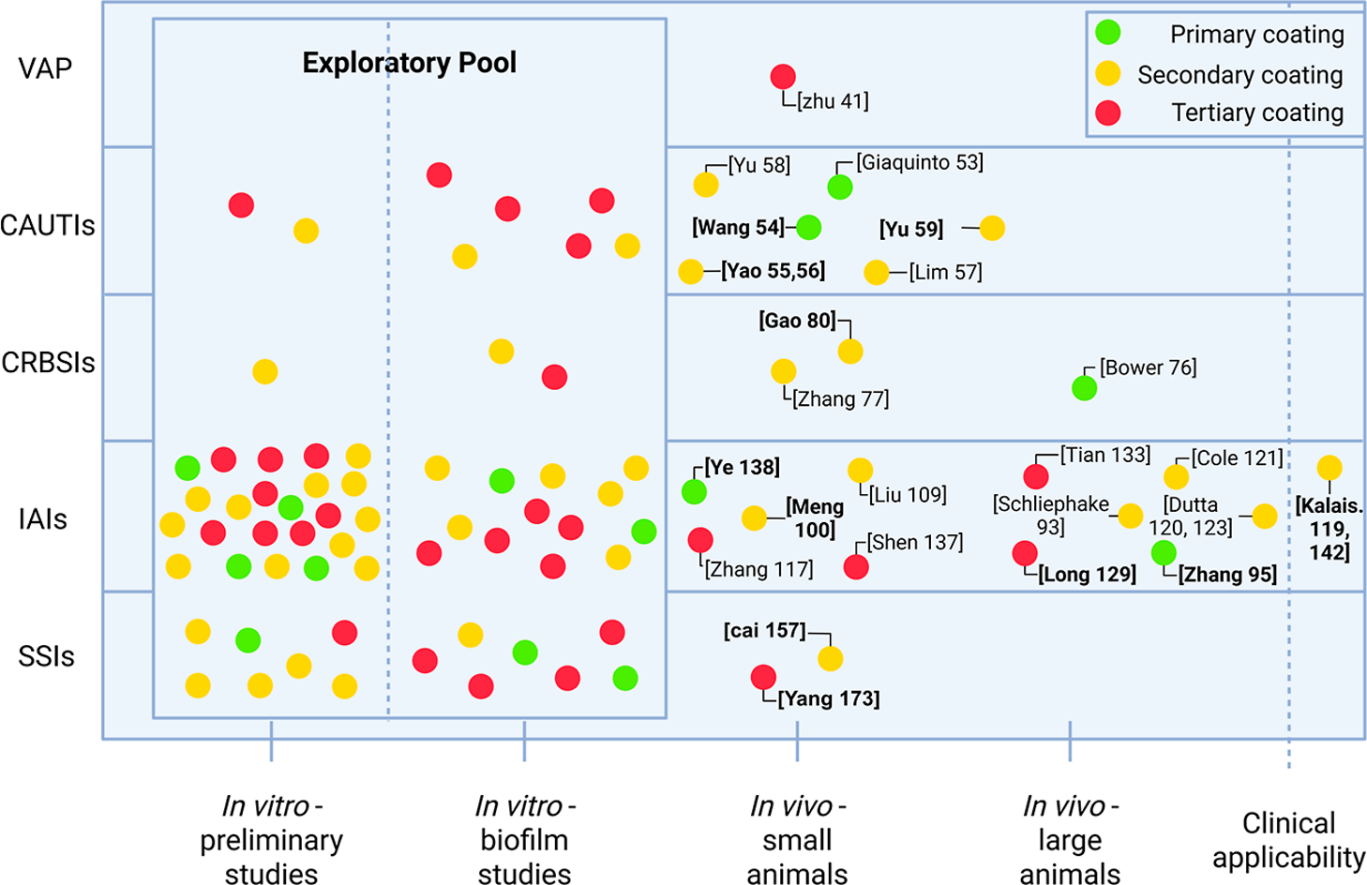
Overview of the translational maturity landscape by pathology, including ventilator-associated pneumonia (VAP), catheter-associated urinary tract infection (CAUTI), catheter-related bloodstream infection (CRBSI), implant-associated infections (IAI) and surgical site infections (SSI). Small animal models include rodent studies with mice and rats, whereas the large animal models refer to studies using rabbits. The most relevant recent publications from 2020 onward are indicated in bold.

Among the most promising CAUTI-preventive coatings are those from Yu et al. and Wang et al., all demonstrating potent in vivo efficacy. Wang et al. developed polymyxin B combination coatings with RRIKA or SAAP159 on silicone catheters, achieving an approximately 3-log reduction of *E. coli* in mouse urine 8 days post infection and preventing bacterial adhesion completely.^54^ Yu et al. designed a PU polymer brush coating functionalized with E6 that reduced *P. aeruginosa* adhesion by > 4 logs and lowered bladder colonization by ±3 logs in mice 7 days after infection.^59^

As a key takeaway for CRBSI prevention, the most promising coatings with in vivo validation include HHC36-functionalized PU tubing of Zhang et al., which exhibited up to a 4-log reduction in *S. aureus* colonization on catheter surfaces 3 days post infection in mice.^77^ Second, Raman et al. reported a β-peptide-modified catheter that markedly suppressed *C. albicans* biofilm formation in a rat central venous catheter model.^50^

Recent studies of AMP-coated implants that have progressed to in vivo stages include those of Long et al., where caerin-F3-coated titanium dental plates significantly reduced bacterial RNA levels in rabbit oral secretions on 7 and 14 days postinfection.^129^ Next, Zhang et al. found that Mel-4 coated titanium plates implanted in rabbits achieved roughly a 1-log reduction in both *S. aureus* and *P. aeruginosa* adhesion 9 days after intravenous infection, indicating moderate suppression of biofilm formation.^95^ Lastly, Ye et al. designed hybrid nanostructures of GLK13 and silver for the coating of titanium implants. The hybrid coating achieved a 2-log reduction of MRSA 4 days postinfection in mouse infection models, outperforming the pure AMP coatings without silver which achieved a 1-log reduction.^138^

Interestingly, several studies report on in human trials of AMP coated contact lenses, including studies focusing solely on biocompatibility, as well as those focused on the prevention of microbial keratitis.^119,124,142,177^ Notably, a recent study by Kalaiselvan et al. reported on a 50% reduction of corneal infiltrative events in participants wearing Mel4-coated lenses compared to the control group in a randomized clinical trial.^119^

Lastly, only a limited number of studies have explored AMP-based strategies for the prevention of SSIs. Cai et al. developed fluorescently traceable antimicrobial sutures functionalized with the K18 peptide via click chemistry, which had a broad-spectrum antibacterial activity on top of their excellent biocompatibility and possibility to study their in in vivo degradation behavior. Yang et al. reported a freeze-dry-thaw microporous hydrogel coincorporating exosomes and AMPs for preventive application.^157,173^ In addition to in vivo antibacterial efficacy, these sponge-like hydrogels exhibited anti-inflammatory, antiapoptotic and regenerative effects on burn wounds.

Although numerous research groups propose innovative coating concepts and generate preclinical data, a pronounced translational bottleneck persists, with only a limited number of coating technologies progressing beyond the preclinical stage. A recurring challenge concerns the transition from promising in vitro and in vivo results to the subsequent phases of medical device development. A primary limitation is the susceptibility of AMPs to degradation in physiological media and their corresponding short half-life in vivo. Consequently, a clear trend has emerged toward the design of (semi)­synthetic AMP analogues rather than naturally occurring gene-encoded sequences. Such modifications not only enhance stability but also enable sequence optimization to improve AMP–surface interactions. Second, this bottleneck is further enhanced by the complexity of the regulatory frameworks for peptide-based drug–device combination products, which exist at the interface of medical devices and regular medicine. This complexity can discourage industrial investment in AMP-based medical devices. Recently however, some guidance to support this process is available through initiatives such as the HAUS program in Korea, the Medicines and Healthcare products Regulatory Agency in the United Kingdom and the U.S. FDA, which provides a dedicated medical device development roadmap.^178–181^ Early alignment with industrial partners has herein emerged as a critical factor in identifying feasibility constraints and guiding coating design toward realistic clinical and commercial implementation.

### 3.8 Prophylactic Peptide Applications Beyond Coatings

Although the prophylactic application of AMPs has been most extensively investigated in the context of antimicrobial coatings on medical devices, alternative delivery formats have been explored. One particular area is oral health, where the rationale derives from the natural abundance of AMPs in the oral cavity, produced by a wide range of resident cell types. Several families of AMPs, including histatins and cathelicidins, are secreted in saliva and oral tissues; however, defensins are considered the principal contributors to maintaining oral homeostasis.^110,182^ Building on this physiological role, both natural and synthetic AMPs have been investigated as promising candidates to support oral hygiene and prevent infections such as dental caries, periodontitis and other mucosal diseases. Notable examples include the synthetic polypeptide GERM CLEAN, formulated as an oral spray, and the peptide C16G2, developed as an oral rinse.^183^ Both are designed to specifically target *Streptococcus mutans* by inhibiting adhesion and suppressing acid production, thereby reducing the risk of caries-associated pathology. In addition, both Tokajuk et al. and Czarnowski et al. have investigated the use of ceragenins for oral hygiene. These studies demonstrated that ceragenins not only exhibit potent activity against pathogenic biofilms but also display reduced cytotoxicity toward host cells when compared with commercially available mouthwashes.^110,182,184^

Next to AMPs in oral hygiene, AMPs are proposed as possible adjuvants in vaccine development. Despite their limited antigenic epitopes and correspondingly weak immune responses, AMPs continue to be investigated as adjuvant components, although their application remains largely conceptual to date.^185^ Some examples of this rationale include the work of Hemmati et al., who used machine learning to develop short immunomodulatory peptides serving as innate immune receptor agonists.^186^ Similarly, Zhang et al. also used an AMP capable of activating the innate immune response via TLR/CCR-like receptors on macrophages.^187^ While holding considerable promise, this technique is still in its infancy and discrepancies between in vitro and in vivo outcomes are frequently observed. Nevertheless, the potential immunomodulatory properties of AMPs warrant careful consideration in the context of future vaccine development.

Lastly, AMPs have been investigated for sepsis prophylaxis owing to their immunomodulatory activity and direct endotoxin-neutralizing properties.^188^

## 4 Conclusion

This review has examined AMPs as preventive strategies against microbial infections and identifies AMP-based surface coatings as the predominant and most extensively investigated application to date. Eradicating bacteria involved in biofilm-associated infections on medical devices is notoriously difficult, much like removing sand from a carpet. Among these medical devices, implants are studied most extensively, as their removal in the event of infection is considerably more complex than for catheter-based devices.

To guide new researchers in the field, Figure 5 outlines a conceptual flowchart for developing prophylactic antibacterial coatings for medical devices. In the first step, a novel AMP is screened and its key characteristics are established. After peptide(s) selection, a wide range of coating strategies is available and selecting the appropriate approach depends on the intended application and the peptide’s properties. A peptide functionalized with a terminal cysteine is well suited for click chemistry; hydrophobic peptides may interact more effectively with polymeric substrates; and peptide length can be critical when designing contact-killing surfaces with regard to their tertiary confirmation and spatial organization after immobilization. Additionally, diffusion requirements must be considered. While many AMPs act through membrane disruption, peptides that rely on intracellular targeting must be released from the coating matrix to exert their effects. As a result, immobilized contact-killing systems must rely on membranolytic AMPs. Furthermore, some tertiary coatings require harsh curing conditions such as UV or heat. Where synthetic peptides tolerate these processes better, natural AMPs often require milder approaches, making layer-by-layer assembly or click chemistry preferable.

**Figure 5:**
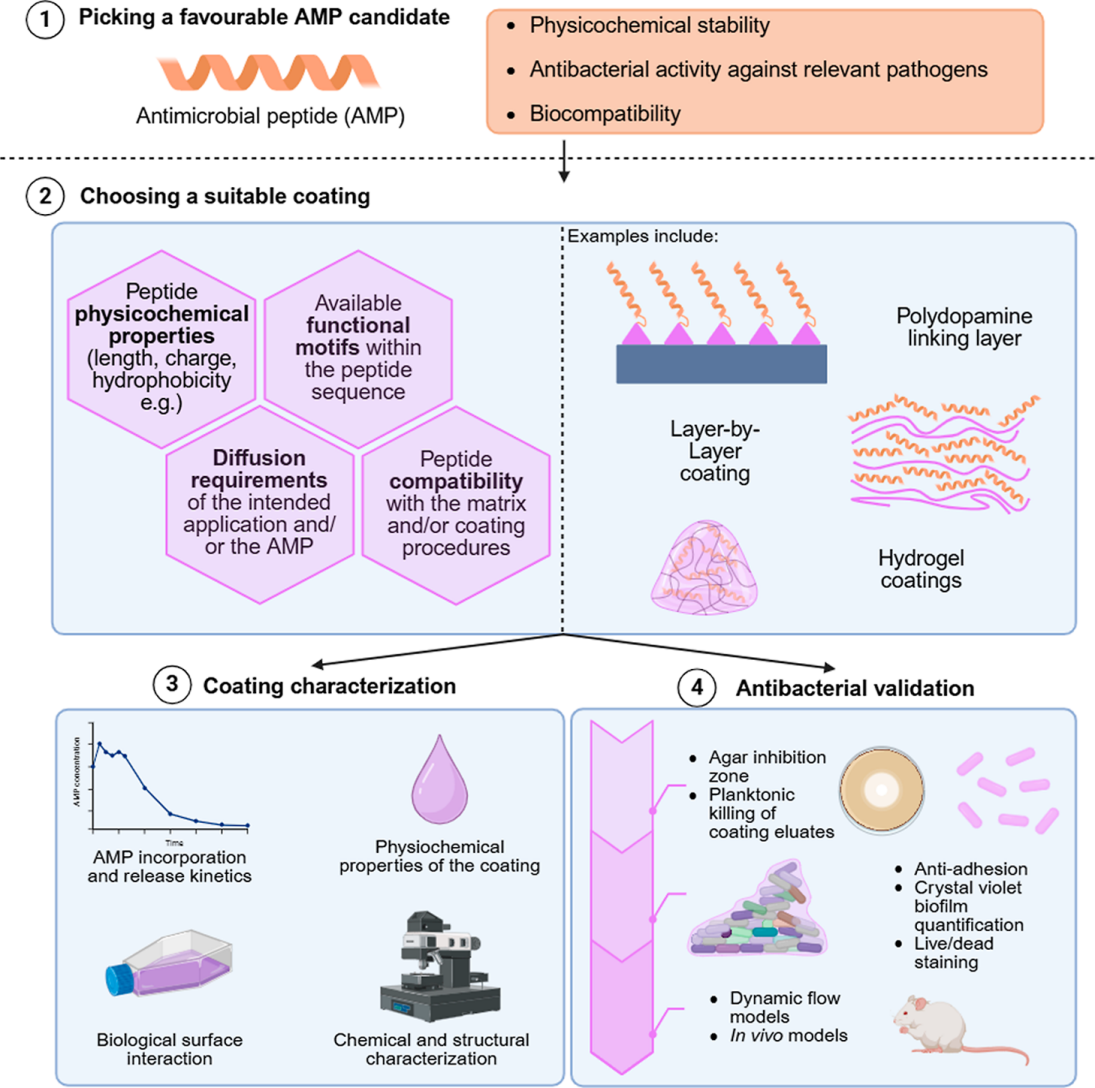
Conceptual flowchart for developing prophylactic antimicrobial surface coatings, from peptide selection to device-level validation.

Beyond peptide selection and coating design, the final material must undergo comprehensive characterization. For diffusion-based systems, AMP release should be quantified (e.g., by high-performance liquid chromatography) and physicochemical features such as wettability and mechanical robustness assessed through water contact angle measurements and adhesion tests. Chemical and structural analysis can be performed using atomic force microscopy or scanning electron microscopy. A final component of coating characterization involves assessing biological surface interactions: mammalian cell adhesion is vital for implants, whereas bacterial adhesion must be minimized across all devices. Antibacterial testing typically progresses from initial planktonic assays, such as eluate-based killing or inhibition-zone measurements, to more relevant antibiofilm studies, for example crystal violet staining. Ultimately, advanced validation requires dynamic flow systems or in vivo infection models. While Figure 5 proposes an experimental flowchart, considerable variability in methodological choices and study prioritization persists across the literature. Focus points for future research include clearer interpretation of ISO 10993-5 biocompatibility guidelines, improved standardization of biofilm assays and their analytical end points, and the inclusion of head-to-head comparisons with clinically relevant reference devices.^189,190^

Although a plethora of research has been conducted with respect to Figure 5, the topic seems to be confined to in vitro or small in vivo studies, with a clear translational bottleneck. This; however, does not imply that the scientific landscape is fully optimized and merely awaiting investment. Continuous innovations and discoveries remain essential, as progress in AMP research is still advancing on a daily basis. Among the most promising emerging strategies is the development of stimuli-responsive hydrogels. These systems exploit infection-associated microenvironmental changes, such as decreases in pH, alterations in enzyme activity, fluctuations in temperature, or the generation of reactive oxygen species, to trigger the controlled release of AMPs from their carriers. Infection-responsive release platforms effectively address several limitations associated with AMP deployment, including premature degradation, limited shelf life and storage stability, and the potential for resistance development. An alternative strategy to advance AMP-based coatings is to shift the focus toward peptidomimetics. These synthetic analogues mimic the structure and function of AMPs while displaying enhanced proteolytic stability and a higher degree of tunability. In addition, their lower production cost makes them appealing alternatives to conventional peptides.

While AMP-based coatings hold substantial promise for preventing DAIs, their translational application remains hindered by intrinsic molecular instability, regulatory complexity and economic constrains. Emerging directions such as stimuli-responsive systems, synthetic analogues or peptide design based on machine learning systems illustrate how rational design can tighten the gap between experimental academic innovation and clinical applicability, moving AMP-based technologies closer to real-world implementation.

## Supplementary Material



## Notes

Pathological-specific PubMed search strings are provided in Supporting Information S1 (PDF)

## Abbreviations

AA: amino acids
AGE: allyl glycidyl ether
AMP: antimicrobial peptide
AMR: antimicrobial resistance
BIP: Bactiguard Infection Protection
CAUTI: catheter-associated urinary tract infection
CDC: center for disease control and prevention
CLS: catheter lock solution
CRBSI: cathether-related bloodstream infection
CVC: central venous catheter
DAI: device-associated infection
ECDC: european center for disease prevention and control
ETT: endotracheal tube
FDA: Food and Drug Administration
HAI: hospital-acquired infection
ICU: intensive care unit
IAI: implant-associated infection
LbL: Layer-by-Layer
MDR: multidrug-resistant
MRSA: methicillin-resistant *Staphylococcus aureus*
PDA: polydopamine
PEEK: polyetheretherketone
PEG: polyethylene glycol
PLGA: poly­(lactic-*co*-glycolic acid)
PVA: poly­(vinyl alcohol)
PVC: polyvinyl chloride
PVP: polyvinylpyrrolidone
SSI: surgical site infection
VAP: ventilator-associated pneumonia
