# Supplementary material for: Antimicrobial Peptides in Preventive Medicine: Current Perspectives on Coating Strategies
Source: ACS Infect Dis. 2026 Feb 24;12(3):978–97. doi: 10.1021/acsinfecdis.5c01050 (PMC13006958; doi:10.1021/acsinfecdis.5c01050)
Supplement: Supplementary file 1 [file id5c01050_si_001.pdf]

Supporting Information

Antimicrobial peptides in preventive medicine: current perspectives on coating strategies

Milan Wouters[†,1], *Laurence Van Moll*[†,1], *Emine Derin* [1,2], *Sara Van Looy* [1], *Linda De Vooght* [1], *Peter Delputte*[1] *and Paul Cos*[*,1]

[1] Laboratory of Microbiology, Parasitology and Hygiene (LMPH), Faculty of Pharmaceutical, Biomedical and Veterinary Sciences, University of Antwerp, Wilrijk, 2000 Antwerp, Belgium

[2] Department of Basic and Applied Science, University of Basilicata, Potenza, Italy

† Both authors contributed equally to this work and were designated as co-first authors

* Author to which corresponding should be addressed

Supplementary information S1: pathological-specific PubMed search strings

Keywords ventilator-associated pneumonia:

> ((VAP[Title/Abstract]) OR (ventilator-associated pneumonia[Title/Abstract])) AND ((coating[Title/Abstract]) OR (coated[Title/Abstract])) AND ((AMP[Title/Abstract]) OR (AMPs[Title/Abstract]) OR (antimicrobial peptides[Title/Abstract]))

Keywords Urinary tract infections:

((Antimicrobial peptide[Title/Abstract]) OR (AMP[Title/Abstract]) OR (peptide[Title/Abstract])) AND ((coating[Title/Abstract]) OR (coated[Title/Abstract])) AND ((Urinary tract infection[Title/Abstract]) OR (urinary tract[Title/Abstract]) OR (UTI[Title/Abstract]) OR (catheter-associated urinary tract infection[Title/Abstract]) OR (CAUTI[Title/Abstract])) AND (catheter[Title/Abstract])

Keywords catheter-related bloodstream infections:

((AMP[Title/Abstract]) OR (AMPs[Title/Abstract]) OR (antimicrobial peptide[Title/Abstract]) OR (antimicrobial peptides[Title/Abstract]) OR (peptide[Title/Abstract]) OR (peptides[Title/Abstract])) AND ((coated[Title/Abstract]) OR (coating[Title/Abstract]) OR (surface-functionalized[Title/Abstract])) AND ((central venous catheter[Title/Abstract]) OR (CLABSI[Title/Abstract]) OR (CRBSI[Title/Abstract]) OR (infected venous catheter[Title/Abstract]) OR (catheter-related infection[Title/Abstract]) OR (catheter-associated biofilms[Title/Abstract]) OR (intravenous catheter[Title/Abstract]))

Keywords implant-associated infections:

((Antimicrobial peptide[Title/Abstract]) OR (AMP[Title/Abstract]) OR (peptide[Title/Abstract])) AND ((coating[Title/Abstract]) OR (coated[Title/Abstract])) AND ((dental implant[Title/Abstract]) OR (joint implant[Title/Abstract]) OR (implant associated infection[Title/Abstract]) OR (valve[Title/Abstract]) OR (orthopedic[Title/Abstract]) OR (orthopedic implant[Title/Abstract]) OR (bone implant[Title/Abstract]) OR (breast implant[Title/Abstract]) OR

(prosthetic[Title/Abstract]) OR (percutaneous implant[Title/Abstract]) OR (lens[Title/Abstract]))

Keywords surgical-site infections:

((coated[Title/Abstract]) OR (coating[Title/Abstract]) OR (surface-functionalized[Title/Abstract])) AND ((surgical site infections[Title/Abstract]) OR (SSI[Title/Abstract]) OR (SSIs[Title/Abstract]) OR (suture[Title/Abstract]) OR (wound dressings[Title/Abstract]) OR (antimicrobial wound dressings[Title/Abstract])) AND ((AMP[Title/Abstract]) OR (AMPs[Title/Abstract]) OR (antimicrobial peptide[Title/Abstract]) OR (antimicrobial peptides[Title/Abstract]) OR (peptide[Title/Abstract]) OR (peptides[Title/Abstract]))
